# Vincristine Chemotherapy Trials and Pharmacokinetics in Tasmanian Devils with Tasmanian Devil Facial Tumor Disease

**DOI:** 10.1371/journal.pone.0065133

**Published:** 2013-06-06

**Authors:** David N. Phalen, Angela Frimberger, Stephen Pyecroft, Sarah Peck, Colette Harmsen, Suzanneth Lola, Beatriz de Mello Mattos, Kong M. Li, Andrew J. McLachlan, Antony Moore

**Affiliations:** 1 Faculty of Veterinary Science, University of Sydney, Sydney, New South Wales, Australia; 2 Veterinary Oncology Consultants, Wauchope, New South Wales, Australia; 3 Tasmanian Department of Primary Industries and Water, Launceston, Tasmania, Australia; 4 School of Animal & Veterinary Science, University of Adelaide, Adelaide, South Australia, Australia; 5 School of Medical Sciences, University of Sydney, Sydney, New South Wales, Australia; 6 Faculty of Pharmacy, University of Sydney, Sydney, New South Wales, Australia; Utrecht University, The Netherlands

## Abstract

Tasmanian Devil Facial Tumor Disease (DFTD) is a transmissible cancer threatening to cause the extinction of Tasmanian Devils in the wild. The aim of this study was to determine the susceptibility of the DFTD to vincristine. Escalating dosage rates of vincristine (0.05 to 0.136 mg/kg) were given to Tasmanian devils in the early stages of DFTD (n = 8). None of these dosage rates impacted the outcome of the disease. A dosage rate of 0.105 mg/kg, a rate significantly higher than that given in humans or domestic animals, was found to the highest dosage rate that could be administered safely. Signs of toxicity included anorexia, vomiting, diarrhea and neutropenia. Pharmacokinetic studies showed that, as with other species, there was a rapid drop in blood concentration following a rapid intravenous infusion with a high volume of distribution (1.96 L/kg) and a relatively long elimination half life (11 h). Plasma clearance (1.8 ml/min/kg) was slower in the Tasmanian devil than in humans, suggesting that pharmacodynamics and not pharmacokinetics explain the Tasmanian devil’s ability to tolerate high dosage rates of vincristine. While providing base-line data for the use of vincristine in Tasmanian devils and possibly other marsupials with vincristine susceptible cancers, these findings strongly suggest that vincristine will not be effective in the treatment of DFTD.

## Introduction

Tasmanian devil facial tumor disease (DFTD) is an aggressive, transmissible and uniformly fatal, malignancy of the Tasmanian devil for which no treatment has been reported [1]. It is one of only two known naturally occurring clonally transmissible cancers (the other being transmissible venereal tumor of canids (CTVT) [2]. DFTD is a monophyletic clonally transmissible tumor whose dissemination appears possible by a down regulation of MHC expression [3], [4]. It is thought to be of Schwann cell origin [5]. It was first described in 1996 and by 2006, it was estimated that 59% of Tasmania was affected by the disease, accounting for population declines of more than 80% in some areas [6]. Older animals have declined as a proportion of the affected population, and the age at breeding has compensatorily declined leading to a shift in life-history of this species [1]. The Tasmanian devil was listed as endangered by International Union for Conservation of Nature in 2009.

Clinically the disease appears as multiple, firm, raised soft-tissue nodules and masses, which are often centrally ulcerated and necrotic. As they progress, they ultimately become large space occupying lesions. The anatomic distribution of DFTD lesions suggests that transmission of transplantable tumor cells occurs during “jaw-wrestling” a common interaction among Tasmanian devils during mating [7]. In most cases, multiple lesions initially arise on the face (especially oral areas) and neck, and it is progressive growth of these lesions that leads to the death of most of the affected animals. While the cause of death for most affected Tasmanian devils is thought to be starvation, metastatic disease occurs rapidly and widely. In affected captive animals the average survival is approximately 3–6 months (our unpublished observation, 2010).

Current management strategies for this disease include isolation of populations of Tasmanian devils by fencing or establishment of isolated peninsulas, and capture and removal of affected Tasmanian devils [8]. Additional “insurance” populations of Tasmanian devils have been established in breeding colonies around mainland Australia. With such reliance on captive populations for survival of the species, investigation into treatment strategies would appear to be important; however none have been reported to date.

Vincristine is effective against a wide range of cancer types due to interference with the mitotic spindle apparatus, causing cell death in mitosis [9]. It has been safely used in a wide range of animal species including chickens, cats, monkeys, dogs, rats, mice and guinea pigs [10]. Vincristine was administered weekly to 201 dogs with CTVT and resulted in a complete remission (CR: 100% tumor reduction) in 197 dogs and a partial remission (PR: >50% tumor reduction but less than CR) in one dog; only one dog relapsed in a period of 12 months [11], [12]. The most common toxicities were mild, self-limiting vomiting or transient leukopenia, seen in less than 15% of dogs; animals older than five years of age were more likely to show gastrointestinal toxicities [13]. Similar toxicities have been seen in cats, and at veterinary dosages used, are considered by most pet owners to be compatible with normal quality of life [14]. In addition to causing a cure in most dogs with CTVT, responses in mesenchymal tumors (sarcomas) have been reported in cats and dogs, implying there may be antitumor efficacy in DFTD [15].

The treatment of wild animals of any species with chemotherapy is probably not realistic, given the potential toxicities and need for supportive care should they arise. On the other hand, the Tasmanian devil is endangered, and it appears that the species may not survive in the wild in sufficient numbers to maintain genetic diversity. With that in mind, the establishment of satellite colonies of Tasmanian devils has now been undertaken. Reliance on relatively small captive populations for the survival of the species means that effective treatment strategies would be very important should DFTD arise in such animals. Additionally, marsupials, in general, appear to be particularly prone to neoplasia [16]. Many are species that are highly endangered and many marsupials are kept in zoos and animal parks creating a need for evidence-based treatment protocols for these species.

The initial objective of this study was to determine if a dosage rate of vincristine could be established that would cause regression of DFTD and produce minimal toxicity in the affected animals. The second objective of this study was to determine the pharmacokinetic parameters of vincristine in Tasmanian devils with DFTD after a single intravenous dose.

## Materials and Methods

### Ethics Statement

All aspects of this project were reviewed and approved by the Tasmanian Government Project Grants Ethics Committee, license AEC Project 40/2007–08.

### Animal Subjects

Wild Tasmanian devils with early stages of DFTD were captured as part of the disease suppression trial and kept in outdoor enclosures with free access to water, food and shelter at the Department of Primary Industry and Water, Launceston, Tasmania. Adult animals with lesions in the early stages of development (defined as 3 or fewer primary lesions, with each tumor measuring less than 4 cm in their widest dimension) were selected for this treatment trial (n = 8) and to be used as untreated controls (n = 8). Animals were acclimatized to captivity for a minimum of one week before the onset of treatment.

### Dosage Escalation Study

The first aim of this study was to determine the optimum dosage rate and interval (minimally effective dose and maximally tolerated dose) for vincristine in Tasmanian devils with DFTD. Vincristine has not been extensively administered to marsupials to the knowledge of the investigators. We expected that marsupials could differ in their risk of toxicity from placental mammals, so rather than rely on scaling from human, dog or cat dosages; a modified Fibonacci dose escalation scheme was employed. Since body surface area dosing has not been found to be reliable in placental companion animals [17], dosage rates were calculated based on body weight (mg/kg). Vincristine (Pfizer, West Ryde, New South Wales) was administered by rapid (less than 1 minute) intravenous bolus injection delivered to Tasmanian devils anaesthetized with isoflurane (Pharmachem, Eagle Farm, Queensland). The dosage was repeated weekly if Tasmanian devils did not show grade 3 or higher toxicity (see below).

The starting vincristine dosage rate was based on dosage rates used in clinical veterinary practice, and previously used safely in a solitary Tasmanian devil by one of us (ASM). The plan was that at each dosage rate, three Tasmanian devils would be treated and evaluated for adverse events. If grade 3 or higher toxicity was not seen then the dosage rate for the next cohort of three animals would be increased according to Fibonacci’s Modified scheme (Table 1). Vincristine was administered by bolus injection through an over-the-needle intravenous catheter placed in either the lateral saphenous, or distal cephalic vein. Catheters were first flushed with 5 mL 0.9% sodium chloride and were again flushed after injection prior to removal using all necessary precautions to prevent human exposure.

**Table 1:** Vincristine dosage rates, numbers of treatments and evidence of toxicity in Tasmanian Devils.

Vincristine Dosage Rate (mg/kg)												
Toxicity (System/Grade)a,b												
Treatment	1	2			3		4	5	6	7	8	9
No.												
Animal												
A	0.05^c^		0.05		0.05							
B	0.05^c^		0.05		0.05		0.07	0.075	0.075	0.105		
							Ax1					
							D1					
C	0.05^c^		0.05		0.05		0.07	0.075	0.075	0.105	0.105	0.136^d^
							Ax1					
										N1		
											An1	
D	0.105		0.105		0.136		0.136	0.105	0.105	0.105		
					Ax1		Ax1	Ax1	Ax3	Ax3		
									D1			
					N1		N3		N2	N3		
E	0.105		0.091^c^									
	Vm1		Vm1									
	D1		D1									
	N2											
F	0.105Vm2D2N4		0.091^c^Vm1D1									
G	0.105		0.091^C^									
	Vm1		Vm1									
	D1		D1									
	N3											

a Toxicity descriptions are for the week following the treatment.

b Abbreviations: Ax – Anorexia, Vm – vomiting, D – diarrhea, An – anemia, N – neutropenia. Numerical values related to toxicity grade.

c Samples were collected for the pharmacokinetic study following administration.

d Animal was euthanized and hematology was not done.

To evaluate toxicity to patients during this trial and to decide if dose escalation was appropriate, previously published criteria were used to evaluate adverse events, where an adverse event was defined as any unfavorable and unintended clinical sign, or abnormal clinicopathologic finding temporally associated with the use of a treatment [18]. These adverse events were graded as to their severity 1 through 5: Grade 1– Mild, Grade 2– Moderate, Grade 3– Severe, Grade 4– Life-threatening or disabling, Grade 5– death (Table 2). General health was assessed by clinical examination, daily assessment of activity, appetite and eliminations, and by a complete blood count including platelet count, serum biochemical profile and urinalysis prior to administering each treatment and then every week when treatments were not administered.

**Table 2:** Criteria for toxic effects in Tasmanian devils receiving vincristine.

Toxic Effect and Grade	Signs
Neutropenia	
0	None
1	1.5–<2.8 neutrophils×10^9^/L
2	1.0–<1.5 neutrophils×10^9^/L
3	0.5–<1.0 neutrophils×10^9^/L
4	<0.5 neutrophils×10^9^/L
Anemia	
0	None
1	25–<31% PCV
2	20–<25% PCV
3	15–<20% PCV
4	<15% PCV
Anorexia/Inappetance	
0	None
1	Inappetance
2	Anorexia <3 days duration
3	Anorexia >3 days but <5 days duration
4	Anorexia >5 days duration; 10% weight loss
Vomiting	
0	None
1	Nausea
2	Sporadic, self-limiting
3	1–5 episodes per day, <2 days
4	6–10 episodes per day, requires hospitalization
Diarrhea	
0	None
1	Soft stools, responds to dietary modification
2	1–4 watery stools per day, <2 days
3	4–7 watery stools per day or >2 days
4	>7 watery stools per day or bloody, requires hospitalization

Supportive care during this trial was limited to providing anti-nausea medications in the form of the parenteral NK-1 antagonist antiemetic maropitant (Cerenia™ Pfizer, West Ryde, New South Wales) at the same dosage rate recommended for parenteral treatment of dogs (1 mg/kg once daily), and the use of a proprietary pet food formulated for palatability and high caloric content (Prescription Diet a/d; Hills Pty Ltd., Topeka, Kansas). In addition the non-steroidal anti-inflammatory meloxicam (Troy Laboratories, Smithfield, New South Wales) was administered parenterally if the lesions appeared inflamed or subjectively painful, and the broad spectrum antibiotic enrofloxacin (Troy Laboratories, Smithfield, New South Wales) was administered to animals with infected lesions.

### Tumor Response and Efficacy of Vincristine

In parallel with determining the optimal dosage rate and interval, we evaluated the efficacy of vincristine in causing remission of spontaneously occurring Tasmanian devil facial tumors. The two greatest perpendicular dimensions of each tumor were measured using calipers, and recorded. Response criteria used in this trial were standard for veterinary oncology trials. A complete response was defined as disappearance of all measurable tumors; partial response was defined as decreased tumor diameters of >50% but <100%; stable disease was defined as a decrease in tumor diameters of <50% or an increase in size up to 25%; progressive disease was categorized as an increase in tumor diameters of >25%, or appearance of new tumor(s). Survival times of animals receiving vincristine chemotherapy, and animals not treated (n = 8) but maintained in identical captivity conditions were compared using the Kaplan-Meier product limit method and Cox regression analysis (SPSS Version 11.0).

The decision to euthanize each treated and untreated animal was a subjective one whose criteria were applied equally to both groups. It was based on assessment of deteriorating quality of life (decreased appetite, reduced activity level and physical changes, including weight loss) co-incident with advanced tumor growth; an example is a fractured mandible that occurred due to tumor invasion. That assessment was done by veterinary staff at the holding facility and animal care attendants who had daily contact with the animals.

### Pharmacokinetic Analysis

Plasma samples to evaluate the pharmacokinetics of vincristine were collected from Tasmanian devils following administration of a single bolus dosage of vincristine. Samples were collected from 6 animals at a vincristine dosage of 0.05 and 0.091 mg/kg (n = 3 at each dose).

All samples for pharmacokinetics analysis were collected through the saphenous catheter that had not been used for chemotherapy administration. Prior to each sample collection a minimum of 0.5 ml of heparin block and blood was withdrawn from the catheter and discarded. After the collection of each sample the catheter was flushed again with heparinized saline (3 mL). Blood samples for measurement of plasma vincristine concentration were collected at 5, 15, 30, 60, 120, 240, 480 min and 23 h after vincristine administration. Animals were anaesthetized for 30 min after treatment; subsequent to that, blood samples were collected through the same catheter with the animal physically restrained. Venous catheters were removed after the 480 min sample collection and blood was drawn directly from the lateral saphenous vein at the 23 h collection. Each blood sample was transferred to heparinized tubes and centrifuged immediately for 10 min. The plasma was harvested and stored at −80°C until analyses.

Plasma samples were analyzed using a modification of previously described and validated HPLC assays for vincristine [19], [20]. Briefly, 250 µl of plasma was spiked with 20 µl of an internal standard, vinblastine, (Velbe, Eli Lilly, Indianapolis, Indiana) in an Eppendorf tube, to give a final concentration of 400 nmol/l. The sample was then diluted with 0.25 ml of 4% phosphoric acid and mixed gently. The SPEC-DAU microdisc SPE cartridges (Varian, Melbourne, Australia) were connected to a Vac Elut and initially conditioned with 0.5 ml methanol, followed by 0.5 ml of 0.1 M phosphate buffer (pH 3.0). Plasma samples were then applied to each cartridge. The sample was allowed to run through the column disc at a low flow rate of no more than 1 ml/min. The cartridge was rinsed with 0.5 ml of 0.1 M phosphate buffer (pH 3.0), followed by 0.5 ml 20% methanol and dried under vacuum. The analytes were eluted with 1 ml of methanol-ammonia (95∶5 v/v) at a low rate of no more than 1 ml/min. The HPLC eluate was then dried under vacuum in a SpeedVac vacuum evaporator (Savant Instruments, Farmingdale, NY, USA) and the dried residue was re-dissolved in 50 µl of mobile phase. The mixture was then vortexed and centrifuged to remove particulates. The supernatant was then transferred to micro insert vials and 10 µl of reconstituted solution was automatically injected into the HPLC system. Plasma vincristine calibration curve of 10–400 nmol/l (9–370 ng/mL) were also prepared similarly and obtained by concentration versus the area ratio of vincristine to vinblastine (IS). The concentrations of vincristine in the unknown samples were calculated from the least-square linear regression equation of the calibration curve.

Chromatographic separation of vincristine and vinblastine (IS) was accomplished using a Waters Symmetry C_8_ 5 µm (2.1×150 mm) micro-bore reverse-phase column (Waters, Rydalmere, Australia) coupled with a 1 mm Opti-Guard C_8_ pre-column (Optimize Technologies, Choice Analytical, Thornleigh, Australia). The mobile phase consisted of a mixture of 10 mM sodium phosphate buffer (pH 6.5), acetonitrile and methanol (45∶35:20, v/v/v). The flow rate was maintained isocratically at 0.3 ml/min. The eluent from the HPLC column was directed via a photo-diode-array (PDA) detector (Shimadzu, Japan) and monitored at 298 nm. The total run time was 15 min.

Median dose-corrected vincristine concentration time data for six animals, three animals at 0.05 mg/kg and three at 0.091 mg/kg, was combined to investigate the pharmacokinetics of vincristine in Tasmanian devils. Pharmacokinetic data were analyzed using non-compartmental methods. The area under the vincristine concentration-time curve until the last time point (AUC_0-t)_) was calculated using the linear trapezoidal rule and was extrapolated to infinity (AUC_0-∞)_) using the last concentration observation (C_t_) divided by the terminal elimination rate constant (k_el_). The terminal elimination rate constant was calculated as the slope of the terminal portion of the natural log transformed concentration-time plot. Half-life was estimated as ln 2/k_el_. The clearance (CL) was calculated as Dose/(AUC_0-∞_) and the volume of distribution (V) was estimated as CL/k_el_.

## Results

### Animals and Dosage Rates

Eight Tasmanian devils with early DTFD were entered into this trial over a period of 27 weeks. The original plan was to only treat animals at a single dosage level, and to escalate dosages only using new cohorts of untreated animals. This was not possible due to limited numbers of animals available for this trial (Table 1). The interval between treatments at the same dosage level was 1 week. When dosage levels were changed in an individual, a minimum break of 3 weeks between treatments was used.

### Dosage Escalation Study

The maximally tolerated dosage rate for a single treatment of vincristine in Tasmanian devils was 0.105 mg/kg. The dose limiting toxicity was neutropenia, with 3 of 5 animals treated at that dosage rate showing grade 2 to 4 neutropenia (Table 1). This would be considered the dosage rate to be used in clinical evaluation. At the next lowest level (0.091 mg/kg) the maximum toxicity was Grade 2 neutropenia in 1 of 4 animals. Only one animal was treated more than once with vincristine at 0.105 mg/kg, and there was a suggestion of cumulative myelosuppression (neutropenia grade 0, week 1; grade 2 week 2; and grade 3 week 3) (Table 1).

### Anti-cancer Activity

Anti-cancer activity was not noted at any dosage rate. All animals treated with vincristine showed either stable disease or progressive disease. All animals treated in this trial, regardless of the dosage of chemotherapy they received, were euthanized as the result of disease progression. There was no decrease in tumor measurements during the trial; stable disease was maintained for between 3 weeks and 9 weeks (median 4.5 weeks) in 6 animals, and PD was noted by 3 weeks in 2 animals (both receiving the MTD or above). The median survival time for the 8 animals receiving vincristine was 129 days, which was not significantly different (p = 0.61) from the median survival time of 83 days for 8 captive Tasmanian devils with DFTD that did not receive chemotherapy.

### Pharmacokinetics

**Figure 1:**
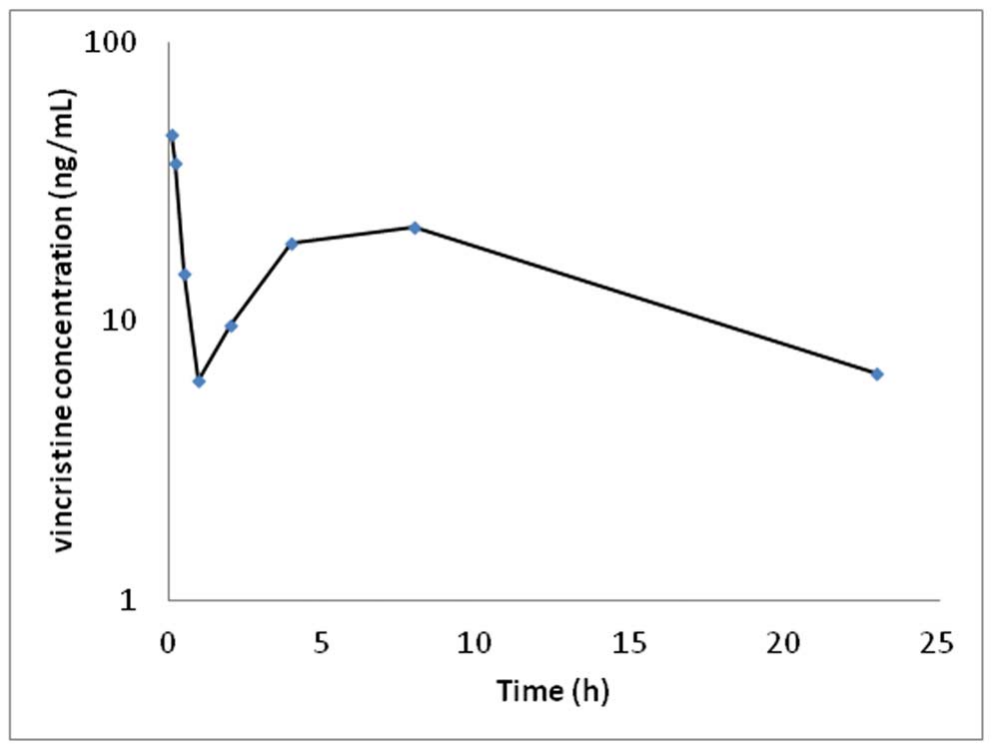
Median plasma vincristine concentration versus time for Tasmanian devils (n = 6) corrected for a dose of vincristine 0.05 mg/kg.

Pharmacokinetic parameters for vincristine in the Tasmanian Devil are presented in Table 3. Figure 1 shows the median plasma vincristine concentration-time profiles of vincristine at a dosage rate of 0.05 mg/kg in the Tasmanian Devil. As expected there was an initial rapid decline in vincristine plasma concentrations, which was consistent with the distribution phase.

**Table 3:** Plasma pharmacokinetic parameters of vincristine in the Tasmanian devil (median data reported corrected for a dose of vincristine 0.05 mg/kg).

Parameter/Dose	0.05 mg/kg
AUC _(0-∞)_ (ng/ml.h)	452.5
t_½_ (h)	11.0
CL (mL/h/kg)	110.5
V (L/kg)	1.76

Note: AUC_(0-∞)_; area under the concentration time curve extrapolated to infinity after a dose of vincristine 0.05 mg/kg, CL;clearance, t_½_; elimination half-life, V;volume of distribution.

## Discussion

If the DFTD was highly responsive to vincristine, then chemotherapy might be a tool that could be used to save animals with important genetic value. In addition, if tumor regression was associated with the development of immunity to the tumor, it might provide an important conservation tool. Vincristine was chosen in this study because, based on clinical experience in cats [15] and dogs [21], it has relatively few side effects, it is effective as a single agent against CTVT and was expected to have limited impact on reproductive capacity of the animals after treatment [12], [22].

Therefore, the initial objective of this study was to determine if a vincristine dosage rate resulting in an acceptable toxicity level would be effective against the DFTD. According to Gehan’s criteria, if no anti-tumor responses are seen in the first 9 animals treated, then there is a less than 10% probability of the relevant chemotherapy drug having a true response rate of more than 25% [23]. We treated 7 animals at a dosage level of 0.105 mg/kg or above with no response. This makes it unlikely that vincristine treatment would have a clinically relevant effect on DFTD in a high proportion of Tasmanian devils. Given that chemotherapeutic drugs that do not have some impact on a tumor when given individually do not potentiate the impact of other chemotherapies, it is also unlikely that vincristine would prove beneficial if combined with other anti-neoplastic drugs.

Neoplastic diseases, in addition to the DFTD, are common in dasyurids and marsupials in general [16]. Therefore, as there are no studies on the use of vincristine in a marsupial, it was the second objective of this study to establish vincristine dosage rates that can be safely tolerated in the Tasmanian devil so that they could be used as reference values for vincristine treatments in other marsupials. Marsupials, including the Tasmanian devil, have metabolic rates that are significantly lower than those for placental animals of comparable body mass [24]. It was therefore expected that vincristine toxicity would be seen at a much lower dosage rate in the Tasmanian devil as compared to that seen in the cat, dog, and human. This did not prove to be true, with the treated Tasmanian devils tolerating a dosage rate (0.105 mg/kg) more than 4 times the dosage rate given to dogs (0.025 mg/kg) [21], 3 times the dosage rate given to cats (0.0375 mg/kg) [15] and more than twice the recommended dosage rate for children less than 10 kg in body weight (0.05 mg/kg) [25].

Pharmacokinetic studies were performed to provide insights into the metabolism and distribution of vincristine in a marsupial and, although unplanned, provided evidence as to why the Tasmanian devils were tolerant of high vincristine dosage rates. Pharmacokinetic studies on vincristine are difficult as there is considerable intra- and inter-subject variability in blood concentrations following the same dosage rates [26]. Also, most studies contain many more study subjects than we were able to enter in this study [26]–[30]. The assay used in this study to analyze vincristine plasma concentration in Tasmanian devils was not sensitive enough (limit of quantification 10 nm/L) to provide late peak concentration for some individuals. As a result a non-compartment pharmacokinetic analysis of pooled data for all six treated Tasmanian devils with DFTD was employed to provide estimates of vincristine pharmacokinetic parameters.

Despite the limitations of the pharmacokinetic study, the results suggest that the pharmacodynamics and not the pharmacokinetics of vincristine explain the Tasmanian devil’s ability to tolerate higher than expected dosage rates. The pharmacokinetic parameters observed in these studies were similar to those seen in humans with an expected rapid initial distribution and a long elimination half-life, consistent with avid and sustained tissue uptake and binding of vincristine. This is further supported by the high volume of distribution (1.76 L/kg) suggesting extensive tissue uptake of vincristine. The elimination half-life in the Tasmanian devil (approximately 11 h) is similar to adult and infant humans [19], [31], [32]. Plasma clearance of vincristine would have been expected to be more rapid than that observed in humans if rapid elimination of the drug played a role in their resistance to toxicity. In contrast, the opposite was observed with the Tasmanian devil having a relatively slower plasma clearance than humans, as might be predicted by their lower metabolic rate. The toxicity of vincristine to tumor cells and presumably to the host cells alike may, in part, be dependent on the concentration that vincristine reaches in the cell. It has been shown that over expression of transmembrane proteins of the ATP-binding cassette (ABC) protein superfamily are responsible for the resistance of some tumors to vincristine [33]–[35]. These enzymes play an important role in normal cells by removing toxic substances from the cytoplasm. It is likely that variability in these enzymes to transport vincristine from cells may occur across species and provides an explanation for why Tasmanian devils are able to tolerate high vincristine dosages.

Some degree of toxicity is an inevitable consequence of chemotherapy, therefore, we documented the toxic effects of vincristine, the clinical manifestations of these effects, and the challenges associated with ameliorating the effects when treating a wild animal. The development of a peripheral neuropathy is the major limiting toxicity for treatment of humans with vincristine [25]. In contrast, gastrointestinal toxicity and bone marrow toxicity limits the dosage rate of vincristine in dogs and cats and intestinal ileus may occur in cats [15] and less commonly in dogs [21]. Neurologic defects were not observed in the Tasmanian devils, however, both bone marrow and apparent gastrointestinal toxicity were.

Neutropenia was the primary hematological toxicity observed, and in the one Tasmanian devil, given more than one dose at 0.105 mg/kg, it appeared to be cumulative. Thrombocytopenia was not seen, and this is consistent with treatment in cats and dogs where vincristine is considered “platelet sparing” [36]. Anemia occurred uncommonly in the treated Tasmanian devils and is not associated with vincristine treatment in other species. Although it could not be determined with certainty, it is likely that this was the result of anemia of chronic disease and blood loss from ulcerated tumors and metastases and was not caused by the vincristine treatment.

Evaluation of the severity of gastrointestinal toxicities seen in the animals in these trials was complicated by the feeding habits of Tasmanian devils and their adjustment to captive living and feeding. Whether the observed reduction in appetite should be considered an adverse event of the chemotherapy is uncertain. All animals were allowed a period to “acclimatize” to captive living, but that needed to be kept brief (usually 1 to 2 weeks) due to the rapid progression of tumor growth, and the need to start treatment before the tumors themselves impacted on their quality of life. Tumor progression and development of visceral metastases often causes appetite loss, and other GI signs; this may also complicate the interpretation of such signs in Tasmanian devils undergoing treatment.

An unanticipated issue in the trial, unique to this species, was maintaining appetite while ensuring asepsis to reduce the risk of infection. Healthy Tasmanian devils in the wild would eat carrion and thus be exposed to heavy loads of bacteria. While this diet is clearly tolerated without ill effect in healthy Tasmanian devils, there was concern that animals debilitated by their disease and further compromised by the vincristine treatments might become septic. Providing a varied diet and using a commercial supplement diet appeared to improve appetite for some animals, but others would only eat carrion. For these Tasmanian devils, macropods that had been killed as part of a culling program and immediately frozen were used as food sources.

The administration of anti-inflammatory drugs, antibiotics and anti-emetic agents, seemed to improve their general well-being during the trial. Specifically, the NK-1 antagonist antiemetic maropitant at the same dosage rate recommended for treatment of dogs (1 mg/kg once daily, orally) appeared effective in the animals in the trial.

### Conclusion

We found no evidence that the DFTD is susceptible to vincristine at subtoxic dosage rates. Tasmanian devils, however, were found to tolerate dosage rates that would have been toxic in dogs, cats, and humans. This tolerance appears to be the result of pharmacodynamic and not pharmacokinetic factors. Marsupials are a diverse groups of animals, and while the data in this paper forms a basis for the use of vincristine in other marsupial species, caution should be taken when treating other species as vincristine metabolism may differ in them.
